# Elevated granzyme B^+^ B-cell level in SIV-infection correlate with viral load and low CD4 T-cell count

**DOI:** 10.1038/icb.2016.96

**Published:** 2016-10-25

**Authors:** Ahmad Kotb, Antonina Klippert, Maria Daskalaki, Ulrike Sauermann, Christiane Stahl-Hennig, Berit Neumann

**Affiliations:** 1 Unit of Infection Models, German Primate Center, Leibniz Institute for Primate Research, Goettingen, Germany; 2 Department of Virology Research, Animal Health Research Institute, Agriculture Research Center, Giza, Egypt; 3 Department of Virology, Faculty of Veterinary Medicine, Cairo University, Giza, Egypt

## Abstract

Granzyme B-expressing (GrB^+^) B cells are thought to contribute to immune dysfunctions in HIV patients, but so far their exact role is unknown. This report demonstrates for the first time the existence of GrB^+^ B cells in SIV-infected rhesus macaques, which represent the most commonly used nonhuman primate model for HIV research. Similar to HIV patients, we found significantly higher frequencies of these cells in the blood of chronically SIV-infected rhesus monkeys compared with uninfected healthy ones. These frequencies correlated with plasma viral load and inversely with absolute CD4 T-cell counts. When investigating GrB^+^ B cells in different compartments, levels were highest in blood, spleen and bone marrow, but considerably lower in lymph nodes and tonsils. Analysis of expression of various surface markers on this particular B-cell subset in SIV-infected macaques revealed differences between the phenotype in macaques and in humans. GrB^+^ B cells in SIV-infected rhesus macaques exhibit an elevated expression of CD5, CD10, CD25 and CD27, while expression of CD19, CD185 and HLA-DR is reduced. In contrast to human GrB^+^ B cells, we did not observe a significantly increased expression of CD43 and CD86. B-cell receptor stimulation in combination with IL-21 of purified B cells from healthy animals led to the induction of GrB expression. Furthermore, initial functional analyses indicated a regulatory role on T-cell proliferation. Overall, our data pave the way for longitudinal analyses including studies on the functionality of GrB^+^ B cells in the nonhuman primate model for AIDS.

## Introduction

Human immunodeficiency virus infection results in a significant dysregulation of T, B and dendritic cells.^1^ Recently, a rare subset of B cells producing IL(Interleukin)-10, called B-regulatory cells (Bregs), was identified in mice and humans.^2^ These Bregs were also demonstrated to produce the serine protease granzyme B (GrB), although GrB usually represents a major key component of natural killer cells and cytotoxic T lymphocytes.^2, 3^ The existence of B-cell-derived granzyme B has been described in the context of infectious or autoimmune diseases, for example, systemic lupus erythematosus,^2^ Sjogren‘s syndrome^2, 3^ and EBV-induced mononucleosis.^2^ So far, the immunological function of these granzyme B-expressing (GrB^+^) B cells remains elusive, and may range from antiviral, cytotoxic to autoregulatory and regulatory functions.^2, 3^ Furthermore, in a recent study, large numbers of circulating GrB^+^ B cells were described in the blood of HIV patients, although this subset is negligible in healthy people.^4, 5^ As studies with HIV-infected individuals are limited regarding routine sampling or collecting samples other than blood, animal studies are needed. To date, the most accepted animal model for HIV research is the experimental infection of rhesus macaques (*Macaca mulatta*) with simian immunodeficiency virus (SIV). To our knowledge, the current study demonstrates for the first time the existence of GrB^+^ B cells in rhesus macaques.

## Results and Discussion

### GrB^+^ B-cell levels are significantly increased in SIV-infected rhesus macaques compared with healthy controls, lack elevated IL-10 expression and correlate with viral load and inversely with absolute CD4^+^ T-cell counts

**Figure 1:**
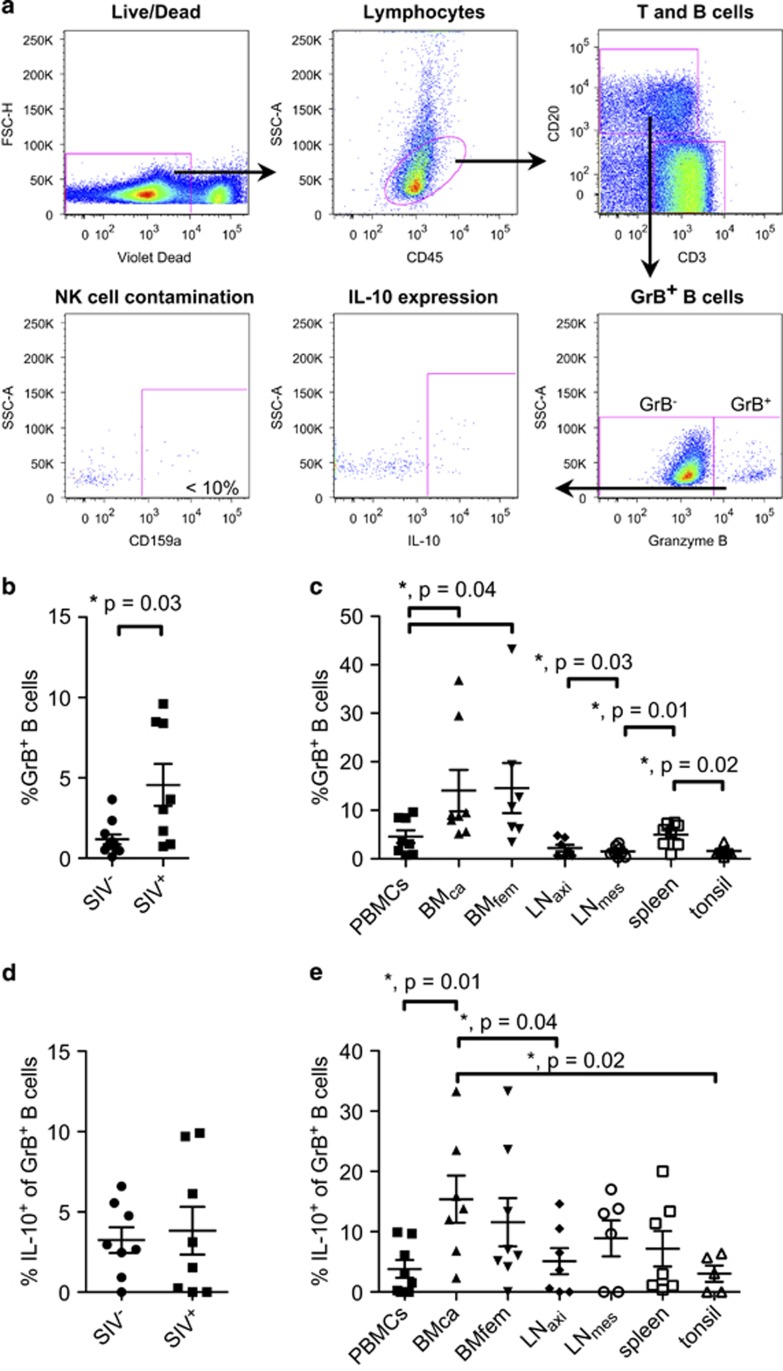
Frequencies of GrB^+^ B cells are significantly increased in SIV-infected rhesus macaques compared with healthy controls. (**a**) Representative gating strategy of GrB^+^ B cells in rhesus macaques. PBMCs of rhesus macaques were isolated and stained for different markers as indicated. Following exclusion of duplets and dead cells, lymphocytes were gated based on CD45 expression. On the basis of CD3 and CD20 expression T and B cells were distinguished. B cells were further analyzed regarding granzyme B expression (GrB^+^ B cells) and expression of IL-10 and the NK cell marker CD159a. (**b**–**e**) Measurements comprised a cross-sectional analysis of SIV-infected macaques 52 weeks post infection (*n*=8) and uninfected healthy controls (*n*=11). (**b**) Frequencies of GrB^+^ B cells in PBMCs of SIV-infected animals compared with healthy controls. (**c**) Frequencies of GrB^+^ B cells in different organs of SIV-infected animals. (**d**) Frequencies of IL-10^+^ of GrB^+^ cells in healthy and SIV-infected animals. (**e**) Frequencies of IL-10^+^ of GrB^+^ B cells in different organs of SIV-infected animals. Analyzed organs are indicated. Error bars indicate s.e.m., *P*-values indicate significance levels.

A representative gating of total granzyme B expression of CD20^+^ B cells for *ex vivo*-derived peripheral blood mononuclear cells (PBMCs) is depicted in Figure 1a. Less than 8% of GrB^+^ B cells expressed the NK cell marker CD159a, indicating a negligible NK cell contamination (Figure 1a, *lower left panel*). We observed significantly higher frequencies of GrB^+^ B cells in blood of SIVmac251-infected animals 1 year post infection compared with healthy controls (SIV^−^: 1.17±1.02% SIV^+^: 4.56±3.69% Figure 1b). This observation is in accordance with a previous study with HIV-infected patients, for which high numbers of circulating GrB^+^ B cells were described.^6, 7^ Additionally, we quantified frequencies of GrB^+^ B cells in different organs of the SIV-infected animals, including bone marrow (BM) and lymph nodes from two different sites, spleen and tonsil (Figure 1c). The highest frequencies of GrB^+^ B cells were detected in BM with no differences between both investigated sites (BMca: bone marrow from iliac crest aspiration): 14.05±12.05% BMfem (bone marrow from femur): 14.56±13.68%, followed by spleen (4.99±2.39%) and PBMCs (4.56±3.69%), whereas the other investigated secondary lymphoid organs analyzed showed lower frequencies (LNaxi (axillary lymph node): 2.22±1.82% LNmes (mesenteric lymph node): 1.51±1.03% and tonsil: 1.59±0.95% Figure 1c). This uneven distribution might be related to the functional properties of GrB^+^ B cells, which are thought to be regulatory.^8^

As GrB^+^ B cells might simultaneously secrete IL-10, we assessed its expression in purified mononuclear cells from blood of healthy and SIV-infected animals as well as from lymphoid organs of SIV-infected animals (Figures 1d and e). We found only a minor portion of GrB^+^ B cells expressing IL-10 in both healthy and SIV-infected rhesus macaques (SIV^−^: 3.24±2.26% SIV^+^: 3.83±4.22% Figure 1d). Of note, highest frequencies were observed at both BM sites (BMca: 15.4±10.4% BMfem: 11.6±11.3%) and mesenteric lymph nodes (11.2±9%), while the other investigated lymphoid organs were comparable to PBMCs (LNaxi: 5.12±5.7% spleen: 7.19±7.74% and tonsil: 3.04±3.03% Figure 1e). The observed low expression of IL-10 indicates that possible regulatory functions of GrB^+^ B cells in rhesus macaques might be mediated by granzyme B itself or other molecules.

**Figure 2:**
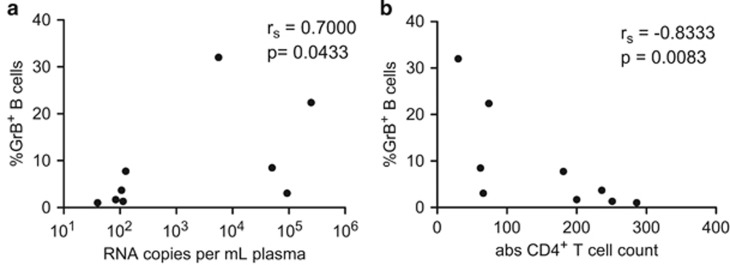
Frequencies of GrB^+^ B cells correlate with markers of SIV disease progression. Blood from *n*=9 SIV-infected animals was collected to determine SIV RNA copy number per ml plasma as well as absolute CD4 T-cell numbers per μl, and frequencies of GrB^+^ B cells at week 46 post infection. Correlation between GrB^+^ B-cell frequencies and (**a**) plasma viral load as well as (**b**) absolute CD4 T-cell counts are shown. *P*-values<0.05 indicate significance (Spearman rank correlation).

In HIV-infected patients, frequencies of regulatory B cells as well as serum levels of GrB correlated positively with HIV viral load, whereas CD4 T-cell counts correlated inversely with these parameters.^9^ We, therefore, sought to analyze the relationship between markers predictive of disease progression and frequencies of GrB^+^ B cells in SIV-infected rhesus macaques. Similar to HIV infection, peripheral GrB^+^ B-cell frequencies correlated significantly with plasma viral load (Figure 2a) and inversely with absolute CD4 T-cell counts (Figure 2b) in samples from SIV-infected animals collected 1 year after infection.

### Phenotypic analyses of GrB^+^ B cells from rhesus macaques revealed differences from reported human GrB^+^ B cells

**Figure 3:**
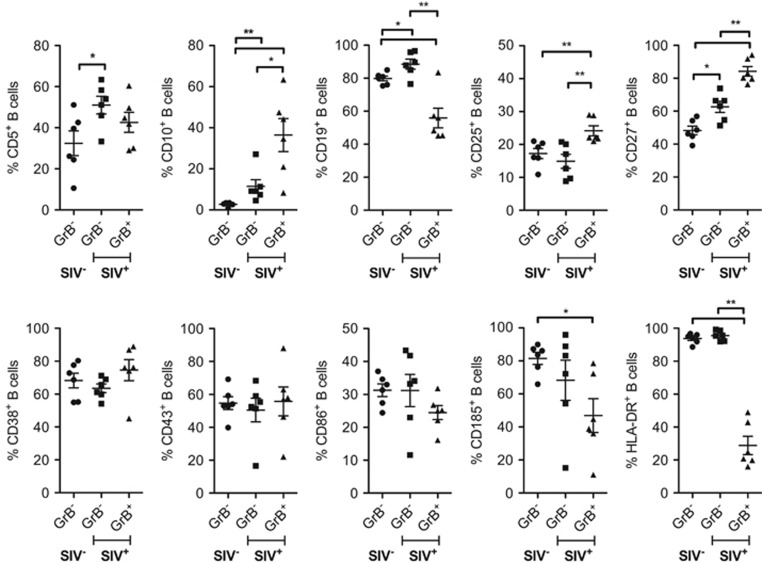
GrB^+^ B cells in SIV-infected rhesus macaques display elevated expression of CD5, CD10, CD25 and CD27, and reduced expression of CD19, CD185 and HLA-DR. The PBMCs from six healthy and six SIV-infected animals were isolated and stained for GrB^+^ B cells as shown in Figure 1a and various surface markers as indicated. Due to the low frequencies of GrB^+^ B cells in blood from healthy rhesus macaques, their phenotypic analysis was not included. Cutoff for the phenotypic analysis of GrB^+^ B cells was set at 1.000 events. Frequencies of positive cells for CD5 (* *P*=0.04), CD10 (*, *P*=0.04; **, *P*=0.002), CD19 (*, *P*=0.04; **, *P*=0.004), CD25 (**, *P*<0.01), CD27 CD10 (*, *P*=0.02; **, *P*<0.01), CD38, CD43, CD86, CD185 (*, *P*=0.02) and HLA-DR (**, *P*=0.002) on GrB^-^ B cells of healthy animals (SIV^−^, *n*=6) as well as GrB^−^ and GrB^+^ B cells of SIV-infected animals (SIV^+^, *n*=6) at week 52 post infection are shown. Error bars indicate s.e.m., *P*-values indicate significance levels.

Despite the observed occurrence of GrB^+^ B cells in various diseases, a specific marker panel is so far missing. We analyzed expression of various markers, including those previously published for human GrB^+^ B cells, in GrB^−^ B cells from healthy rhesus macaques as well as GrB^−^ and GrB^+^ B cells from SIV-infected animals (Figure 3). Overall, GrB^+^ B cells in SIV-infected rhesus macaques show an enhanced expression of CD5, CD10, CD25 and CD27, and reduced expression of CD19, CD185 and HLA-DR, when compared with B cells of healthy animals (Figure 3).

The observed phenotype of GrB^+^ B cells in SIV-infected rhesus macaques corresponded only partially with the reported one of their human counterparts. GrB^+^ B cells in HIV-infected patients show a significantly increased expression of CD5, CD10, CD43 and CD86, but a significantly reduced CD27 expression.^10^ The remarkably low HLA-DR as well as high CD27 expression observed in GrB^+^ B cells of SIV-infected rhesus macaques implicates a regulatory function of these cells.

### First insights into functional properties of GrB^+^ B cells

**Figure 4:**
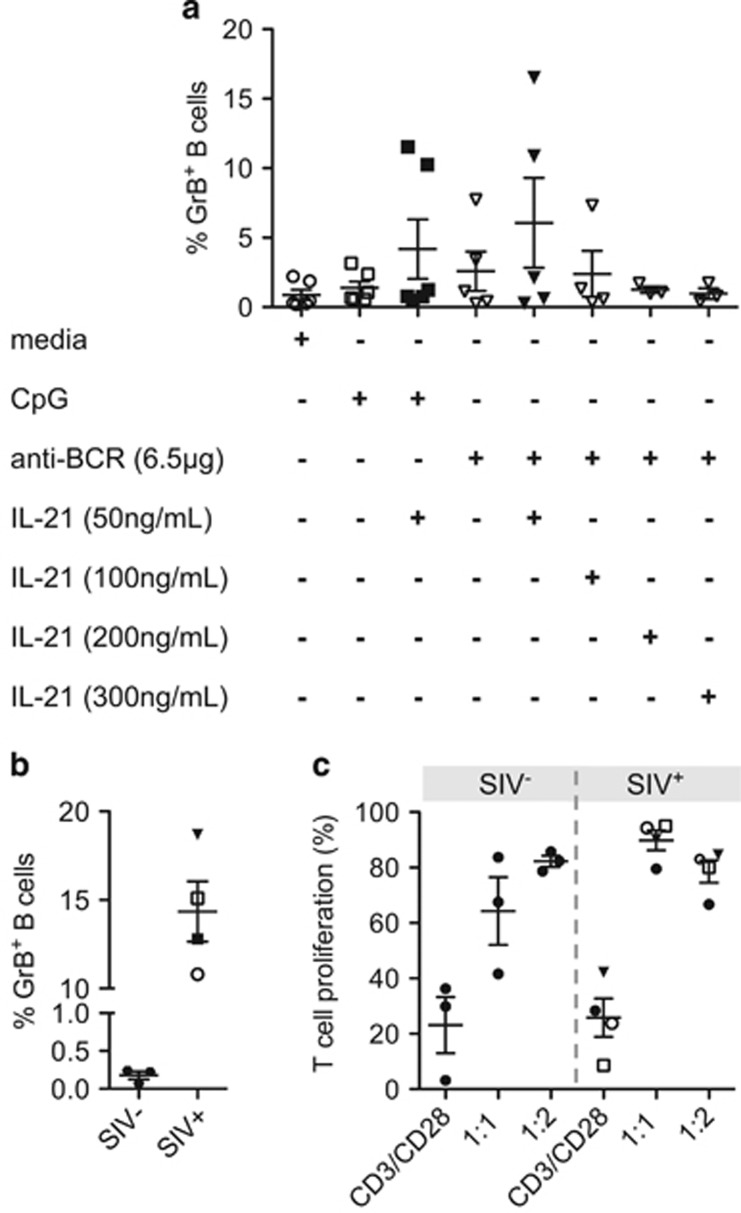
Functional properties of GrB^+^ B cells (**a**) Purified CD20^+^ B cells isolated from healthy animals (*n*=6; mean values of up to four measurements per animal are shown) were cultured for 48 h in the presence or absence of IL-21, CpG and anti-BCR as indicated, stained and analyzed by FACS. Frequencies of GrB^+^ B cells upon B-cell stimulation are shown. (**b** and **c**) influence of GrB^+^ B cells on autologous CD4 T cell proliferation. (**b**) Frequencies of GrB^+^ B cells in healthy (SIV^−^, *n*=3) and SIV-infected animals (SIV^+^, *n*=4). (**c**) CD4^+^ T cells of healthy and SIV-infected animals were purified, CFSE-stained and stimulated with plate-bound CD3 and CD28 to induce proliferation. T cells were cultured alone or with autologous B cells at ratios of 1:1 and 1:2. After 6 days, proliferation of CD4^+^ T cells was analyzed by FACS. Percentage of proliferating T cells (CFSE^low^) are shown.

*In vitro* stimulation of both healthy and malignant human B cells with IL-21 and antibodies raised against the B-cell receptor (anti-BCR) results in the induction of GrB expression in these cells.^6, 7, 11^ We tested different combinations of B-cell stimulants (CpG and anti-BCR) and increasing concentrations of IL-21 for their capacity to induce GrB expression in magnetically purified CD20^+^ B cells of healthy rhesus macaques (Figure 4a). Frequencies of GrB^+^ B cells were substantially increased in two of six animals upon stimulation with 50 ng ml^−1^ IL-21 in the presence of CpG or anti-BCR (11–16.5%) when compared with unstimulated cells (about 2%). In the other animals, a two-to five-fold-induction of GrB expression was observed (media: 0.29±0.1% CpG: 0.71±0.3% and anti BCR: 1.25±0.39%). Neither increasing amounts of IL-21 and anti-BCR nor a longer period of stimulation resulted in a higher induction of GrB expression (data not shown). As reported for humans, B-cell stimulation in combination with IL-21 also led to the induction of GrB expression in rhesus B cells. Still, further and improved studies are needed as percentages of GrB^+^ B cells were still quite low.

To gain first insights into the functional properties of GrB^+^ B cells, we aimed at analyzing their effect on autologous CD4^+^ T-cell proliferation. Therefore, we magnetically purified CD20^+^ B cells as well as CD4^+^ T cells of four SIV-infected animals with high GrB^+^ B-cell frequencies and compared obtained data with those of three healthy rhesus macaques (SIV^−^: 0.18±0.09% SIV^+^: 14.35±3.39%, Figure 4b). To induce T-cell proliferation, CFSE-stained CD4^+^ T cells were stimulated with plate-bound CD3/CD28 antibodies (% proliferation SIV^−^: 23.3±17.6% SIV^+^: 14.35±3.39%, Figure 4c) and incubated alone or in the presence of autologous B cells at ratios of 1:1 and 1:2. While incubation at a ratio of 1:1 enhanced T-cell proliferation in both healthy and SIV-infected macaques (SIV^−^: 64.3±21.2% SIV^+^: 89.8±7.2%, Figure 4c), double amounts of B cells led to a reduced proliferation in case of SIV-infected macaques, although not reaching significance (SIV^−^: 82.3±3.5% SIV^+^: 78.6±8.1%, Figure 4c). These data give first hints about a regulatory function of GrB^+^ B cells on T-cell proliferation.

It was proposed that HIV infection leads to incompletely activated T cells still capable of normal IL-21 secretion, but reduced expression of CD40L.^11^ Such T cells may lead to the induction of GrB expression in B cells. We also observed a similar IL-21-expression in healthy and SIV-infected rhesus macaques, while expression of CD40L was significantly reduced in SIV-infected animals (data not shown), indicating similar mechanisms for GrB induction in B cells in SIV and HIV infection.

In summary, we showed increased frequencies of circulating GrB^+^ B cells in SIV-infected rhesus macaques that correlate with markers prognostic for disease development. We also provided frequencies of these cells in primary and secondary lymphoid organs of infected animals as well as their phenotypic characterization. Furthermore, first functional analyses were performed. This study highlights for the first time the relevance of granzyme B-expressing B cells in SIV-infected rhesus macaques and provides an excellent starting point for subsequent analyses regarding their functionality.

## Methods

### Animals

Indian-origin rhesus macaques (*Macaca mulatta*) were housed at the German Primate Center under standard conditions complying with §§7–9 of the German Animal Welfare Act, which strictly adheres to the European Union guidelines (EU directive 2010/63/EU) on the use of non-human primates for biomedical research. The experiments were approved by an external ethics committee of the Lower Saxony State Office for Consumer Protection and Food Safety. Nine animals (seven males and 2 females, 6–9 years) were inoculated intrarectally with 120 TCID_50_ SIVmac251. Blood and organ samples were collected 46 or 52 weeks post infection and organ samples upon necropsy at week 46 and 52 post infection. For CFSE proliferation assay, PBMCs from four rhesus macaques infected with SIVmac239 or -251 were used, including one progressor (female, 6 years, 74 weeks post infection) and three long-term non-progressors (male, 11–15 years, infected for 5–10 years). Blood samples of a total of 11 healthy rhesus macaques were analyzed (10 males, 1 female, 5 –12 years).

### Isolation of cells, flow cytometry, stimulation and cell analysis

Tissue sampling, processing and flow cytometric analysis were performed as described previously.^11^ Used monoclonal antibodies for the detection of GrB^+^ B cells as well as their phenotypic characterization are listed in Supplementary Table 1. CD20^+^ B cells and/or CD4^+^ T cells from PBMCs were purified (>96% purity) using appropriate MACS kits (Miltenyi Biotec, Bergisch Gladbach, Germany). B-cell stimulation and CFSE proliferation assay were performed as described previously.^7^ To induce T-cell proliferation, plate-bound CD3 and CD28 antibodies (both from BD Biosciences, Heidelberg, Germany) were used.

### Plasma SIV RNA levels

Viral RNA levels were quantified by real-time RT-PCR assay as previously described.^11, 12^

### Statistical analysis

Means±s.e.m. were calculated using GRAPHPAD PRISM version 5.0 for MAC OS 10.9, GRAPHPAD Software, La Jolla, CA, USA. The Mann–Whitney test was applied to compare the groups and the Spearman rank test to assess potential correlations. Differences were considered to be statistically significant when *P*<0.05.
