# Supplementary material for: Elevated granzyme B+ B-cell level in SIV-infection correlate with viral load and low CD4 T-cell count
Source: Immunol Cell Biol. 2016 Oct 25;95(3):316–20. doi: 10.1038/icb.2016.96 (PMC5364320; doi:10.1038/icb.2016.96)
Supplement: Supplementary Information [file icb201696x1.pdf]

Supplementary Table I: Used Antibodies for the identification and phenotypic characterization of GrB^{+} B cells in rhesus macaques

**Antigen**	**Clone**	**Format**	**Company**
CD3	SP34-2	AF700	BD
CD5	UCHT2	PE	BL
CD10	HI10a	APC-Cy7	BL
CD19	J3.119	PE	BC
CD20	2H7	PE-Cy7	BL
CD25	M-A251	APC-Cy7	BL
CD27	O323	BV650	BL
CD38	OKT10	APC	NIH
CD43	DFT1	FITC	BC
CD45	D058-1283	V500	BD
CD86	IT2.2	AF488	BL
CD159a	Z199	APC	BC
CD185	MU5UBEE	APC	E
HLA-DR	L243	APC-Cy7	BL
Live/dead		V450	I
Granzyme B	GB11	PE-CF594	BD
IL-10	JES3-9D7	APC	BL

APC = allophycocyanin; AF = Alexa Fluor; BV = Brilliant Violet; Cy = Cyanin; FITC = fluorescein isothiocyanate; PE = phycoerythrin;; BC, Beckman Coulter, Krefeld, Germany; BD = BD Bioscience, Heidelberg, Germany; BL = BioLegend, San Diego, CA, USA; E = eBioscience, San Diego, CA, USA; I = Invitrogen; Karlsruhe, Germany; NIH = The U.S. National Institute of Health Nonhuman Primate Reagent Resource, Boston, Ma, USA; SB = Southern Biotech, Birmingham, AL, USA
